# Actual and symbolic prisons, Black men, and the freedom-unfreedom paradox: interrogating the bad faith of racialized oppression in a post-accountable United States

**DOI:** 10.3389/fpsyg.2023.1235185

**Published:** 2023-10-09

**Authors:** Darron T. Smith, Brenda G. Harris

**Affiliations:** ^1^University of Washington, Seattle, WA, United States; ^2^Department of Teacher Education, Southern Utah University, Cedar City, UT, United States

**Keywords:** race, freedom, masculinity, symbolic, Blackness

## Abstract

Drawing on systemic racism theory, white racial framing and the notion of bad faith as the connecting, justifying thread between ideals of freedom and equality and actions realizing unfreedom and inequities, this essay explores the alchemy of race, masculinity, and racialized oppression and its consequences for Black men past and present in United States society. This essay’s aim is to trace the historical ideologies and cultural practices, relations, and normative standards that have contributed to, and hence must be challenged to confront, the inequitable, race-based relations of power, and privilege at the root of institutionalized injustices. To do so, this essay interrogates the dissonance of bad faith as a corrective mode of truth telling to highlight and tap the equity potential of Black men’s collective, historical rejections of the White mainstream’s conflicting definitions and deceptive reasonings requisite for pushing toward racial justice, healing, and peace.

## Introduction

And, in fact, the truth about the Black American man, as a historical entity and as a human being, has been hidden from him, deliberately and cruelly; the power of the white world is threatened whenever a Black American man refuses to accept the white world’s definitions. So every attempt is made to cut that Black American man down—not only was made yesterday but is made today (Baldwin, 1962/2021, p. 69).

In the epigraph above, American writer and cultural critic points of Baldwin (1962/2021) to Black men, and their role in United States society, as embodying a threat to the existing racial hierarchy so significant that patterned violence against them at the hands of representatives of the White mainstream has historically been viewed as officially necessary for the public good and thus required no justification. The May 25th murder in 2020 of Mr. George Floyd, a 46-year-old unarmed Black man, by a White police officer, Derek Chauvin, is but one example of a Black man deemed a threat significant enough by the *white world* to merit being cut down with life-ending violence at the hands of official representatives of that *white world* referred to by Baldwin (1962/2021); Floyd lost his life while handcuffed as the now former police officer Chauvin knelt on Floyd’s neck for nearly 10 min (Moody-Ramirez et al., 2021).

Beyond Mr. Floyd, as a visit to “The Legacy Museum: From Slavery to Mass Incarceration” in Montgomery, Alabama (Pierce and Heitz, 2020) captures, there are voluminous recent and less remembered examples of Black men deemed threats to United States society’s White mainstream (Whitestream) and cut down by White violence for refusing to accept the *white world’s definitions* (Baldwin, 1962/2021). To say the names of only six other Black men also who likewise have more recently gained unwanted membership in this race-based group built on White violence (Cottman et al., 2023): Ahmaud Arbery, Daunte Wright, Jordan Neely, Tamir Rice, Trayvon Martin, and Jawan Dallas (Nicholson et al., 2009; Weissinger et al., 2017). That whole groups of people must take to the streets to proclaim “Black Lives Matter” demonstrates the enduring veracity of points of Baldwin (1962/2021) about Black men and their historical role in United States society (Szetela, 2020).

Manifested by the historically, racially patterned murders of unarmed Black men, then, the bodies of Black men in United States society have consistently served as primary targets and repositories of the harms and traumas realized by the deeply systemic, inequitable allocations of power, distribution of valued resources, and consequent abuses incurred by representatives of its Whitestream across generations (Ferreira da Silva, 2009; Feagin and Ducey, 2018). What is it about Black men that makes them a consistent threat deemed so significant to United States society’s Whitestream that every effort continues to be made to cut them down using racial violence, for the public good no less (Patterson, 2018; Wilderson, 2020)?

As Baldwin (1962/2021) explains above, it is the violence itself of United States society against Black men that is a site rich with potential answers to this question of the Whitestream’s enduring use of Black men as a historical entity and human beings; the patterned, state-sanctioned violence, including threats of violence, against the bodies and lives of Black men are more than simple reactionary responses to racial prejudices, biases, and antipathy for Blackness (i.e., characteristics, aims, goals, histories, and accomplishments associated with Black people; Kelley, 1997; Mills, 1997; Bonilla-Silva, 2003). Rather, the very degree of the Whitestream’s rage and perceived need for elimination-oriented violence against Black men is itself a red flag marking the immensity of the collective potential of Black men to confront and transform United States society’s existing racial status quo privileging Whiteness (i.e., characteristics, goals, interests, values, and histories associated with White people as a group) toward more humanized, participatory social spaces and relations (Yancy and Alcoff, 2016; Feagin, 2020).

Specifically, Baldwin (1962/2021) above directs us to answers by highlighting the effects of historical power asymmetries. First, he emphasizes the Whitestream’s lethal historical subjugation of Black men in the United States. Next, he emphasizes the dynamic, immense power Black males wield and are endowed with the capacity to challenge White racial dominance. Then, he argues that the severity of White people’s violence against Black males is proportional to the power of Black men to confront institutionalized racism and inequality; in short, the degree of United States society’s rage and violence against Black men in society matches the degree to which Black men collectively threaten to disrupt and overturn the patterned imbalances of power and access to resources and opportunities privileging White people collectively across its valued institutions.

If, as Baldwin (1962/2021) observes, above and as the degree of United States society’s patterned violence against Black men and their bodies indicate, Black men collectively share this immense transformative potential for humanized living and the disruption of historical imbalances of power and abuses, then how can this equity potential be tapped toward racial healing, peace, and the thriving and wellbeing of individuals and groups and against the patterned oppressions so many continue to endure? Put simply, how is that raced, gendered equity potential constituted today and how can it be applied?

Drawing on systemic racism theory (Feagin, 2006), White racial framing (Feagin, 2020), and the notion of bad faith (Gordon, 1995, 1997) as the connecting, justifying thread between ideals of freedom and equality and actions realizing unfreedom and inequities, this essay explores the alchemy of race, masculinity, and racialized oppression and its consequences for Black men past and present in United States society (Hooks, 2004b; Yancy, 2022). This essay’s aim is to trace the historical ideologies and cultural practices, relations, and normative standards that have contributed to, and hence must be challenged to confront, the inequitable, race-based relations of power and privilege at the root of institutionalized injustices (Tichavakunda, 2021). To do so, this essay interrogates the dissonance of bad faith (Gordon, 1995, 1997) as a corrective mode of truth telling to highlight and tap the equity potential of Black men’s collective, historical rejections of the Whitestreams’s conflicting definitions, and deceptive reasonings requisite for pushing toward racial justice, healing, and peace (Lozenski, 2016).

## The freedom-unfreedom paradox, oppression, bad faith, and healing

The United States has always viewed itself as exceptional (Foner, 1999). And, indeed, the United States is exceptional—exceptional as a nation that incarcerates more of its citizens than any other country in the world (Alexander, 2010,2020). How is it that in this land of freedom, entire groups of people feel compelled to take to the streets regularly to assert that their lives matter and that they should not be shot and murdered arbitrarily by police? These apparent contradictions filled with violence reflect American society’s longstanding paradox of official, full-throated freedom (for some) coupled with disavowed, racialized unfreedom (faced by minoritized others; Mills, 1997), a kind of herrenvolk freedom (Glaude Jr, 2017).

This seeming contradiction between freedom and unfreedom, in turn, is a manifestation of systemic oppression based on bad faith. Systemic oppression refers to the patterned systems of practices, norms, rules, methods, laws, and relationships that uphold and reinforce the Whitestream’s collective group position.

Bad faith informs, operationalizes, and manipulates racial dominance and other difference-based forms of oppression (e.g., sexism, ableism, classism, homophobia, etc.; Gordon, 1997). Bad faith denotes unconscious or intentional self-deception; the term refers to people’s tendency to lie to themselves to protect their feelings, avoid having to take corrective action, and maintain a positive self-image (Gordon, 1995); it also has legal connotations used to describe intentionally dishonest acts committed within contractual obligations (Tichavakunda, 2021). Importantly, bad faith prevents critical dialogue by purposely or unintentionally evading the truth, choice, and responsibility in favor of deceptive, selective memory, falsehoods, and distortions.

## Theorizing systemic oppression and its connections to bad faith

Systemic racialized oppression is rooted in bad faith; it is sanctioned by bad faith; and it is an expression of bad faith. To comprehend and then combat systemic oppression, it is necessary to identify and interrupt the logic of bad faith that binds the apparent paradox of freedom and unfreedom in United States society.

In today’s ostensibly post-racial society, the notion of being accused of racism or labeled a racist offends most White Americans. Racism, for many White Americans, is synonymous with racial extremists on the fringes of society (e.g., neo-Nazis); racists are “those” individuals who don white hoods and swastikas tattooed on their arms--individuals who are appropriately far removed from the mainstream of society (Smith, 2005). From this vantage point, race and prejudice are associated with individuals’ seemingly irrational actions, thoughts, and decisions; accordingly, from this perspective, friendships with individuals from minoritized groups, then, are sufficient as evidence of anti-racism and good moral character among White people (Jackman, 1986).

To eradicate oppression, however, there is no precedent for correcting people’s flawed reasoning (DuBois, 1899/2023; Fanon, 2013). Furthermore, systemic racial oppression is manifested at both the macro- and micro-levels of society (Feagin, 2006, 2020). White superiority is enacted by individuals and groups socialized to apply White racial framings of society to make sense of and guide their actions, interactions, and decisions based on the epistemological reasoning that White ways of knowing and being are superior to those of others (Arendt, 2020).

## Unfreedom and Black male bodies

As Baldwin (1962/2021) indicates above, White domination over the Black body has been relentlessly pursued throughout United States history, both physically and symbolically with Black men symbolically demonized as criminal and dangerous and thus negative imagery providing some justification for enacted physical containment of, and violence against, their bodies (Dean, 2000; Wacquant, 2000). Indeed, in the United States, Black men and boys continue to be unfree at much more acutely concentrated rates as compared to their White counterparts (Alexander, 2010,2020); in 2019 at midyear, the incarceration rate for Black people in the United States held steady at 600 per 100,000 individuals incarcerated, a rate more than three times the rate for White people (Zeng and Minton, 2021). Since 2015, the daily incarceration rate for Black men ages 16–34 is 9.1 percent of their identity group or population as compared to 1.6 percent of the population of young White men, and 3.9 percent of the population of young Latinx men (Pettit and Gutierrez, 2018).

Nonetheless, one need not be physically confined to be imprisoned—one’s body behind constraining iron bars; symbolic incarceration is likewise a lived realty for far too many Black males in United States society’s Whitestream, a type of imprisonment of the mind that then imprisons the body despite lack of any physically constraining iron bars (Turner, 2019; Lomotey and Smith, 2023). While fatality rates continue to decline among men of other races and ethnicities, they continue to rise among Black men (Mutua, 2006; Dodson, 2021). With Black men consistently represented by United States society’s Whitestream as dangerous and potential and budding threats, gun violence is increasingly the primary contributor to the high death rates among Black men (Benjamins and De Maio, 2021). Among males aged 10–25, Black-on-Black homicide is a significant public health crisis as negative images, symbols, and representations are linked to cultural practices and internalized and acted on by larger society and targeting Black men (Kaiser Family Foundation, 2006; Sum et al., 2009; Zimring, 2017).

Similarly, the rate at which Black male students are suspended and expelled from public school is three times that of their White peers (Skiba et al., 2000; Bottiani et al., 2018). Despite more than 50 years of racial desegregation and school reform efforts (Boyd, 2009), Black male students are routinely punished more severely, assigned to lower-ability groups, and expelled at a higher rate than their white counterparts (Bradshaw et al., 2010). Seen as likely, emerging, and existing problems or dangerous to others, especially White students, Black male learners are consistently taught by teachers who would prefer not to work with them (Irvine, 1990; Fuller, 1992; Delpit, 1995; Hale, 2001) and subjected to harsher disciplinary practices as compared to their peers in all other racial and ethnic groups (Voelkl et al., 1999; Noguera, 2003; Cook et al., 2018).

The well-documented relationship between United States public schools and prisons creates a “deadly symbiosis” (Wacquant, 2000) between education and the incarceration of Black males (King, 2005; Kim et al., 2010; McIntosh et al., 2021). How does this unfreedom of Black men persist in the land of the free (Bureau of Justice Statistics, 2014; Reed et al., 2014)? How does bad faith transform over time to justify and sustain society’s paradox of freedom and unfreedom as it mutates into ever varying forms and configurations?

## Tracking bad faith historically: European contact and race

The dominant ideologies of Black males as subhuman, violent, criminal, and bad are indications of deeply rooted bad faith (Grant, 2019); bad faith logic enables the Whitestream to maintain a pleasing collective self-image while perpetuating violence against any threat to this pleasing self-image of superiority (Tichavakunda, 2021).

Because evidence is superfluous within the logic of bad faith (Gordon, 1995), these dominant negative ideologies regarding Black people and Black males especially persist across generations (Sullivan and Cross, 2016). No quantity of counterevidence is sufficient to refute anti-Black prejudice. The first contact between Europeans and Africans, during which Africa’s natural and human resources were violently exploited, is the source of much of the anti-Black (mis) information used to create the perception of Black unfreedom in a free nation (Karenga, 2002).

Specifically, Western colonial powers created race-based distinctions according to skin color, hair texture, genitalia, facial features, and other phenotypical characteristics to differentiate themselves from Africans and justify violent oppression (Rodney, 1972). Through this White racial lens (Feagin, 2020), Black people were placed at the bottom of a human hierarchy based on their “thick lips, ““flat noses, “and “wool-like hair, “among other physically discernible characteristics (Gossett, 1997).

On a biological level, these racial distinctions were thought to control Black people’s conduct and moral foundations (Jordan, 1974). This belief has persisted for centuries (Hall, 1997; Montagu, 1997; Brook, 1999). Biological determinism, still common today, classified Black males as childish, criminals, oversexualized, and more likely to commit offenses (Smedley, 1993; Smith, 2005). This belief held that these characteristics were inborn qualities that required physical punishment, subordination, and containment for the purpose of correction and the protection of society (Dean, 2000; Said, 2003).

United States society has a long history of sending distorted messages of low regard and mistruth to Black men and boys (e.g., Brinton, 1890; Watkins, 2006; Croteau and Hoynes, 2014). Although they change form over generations, these negative images of Black men and Blackness are consistently used for the same purpose over time—to justify Black males’ symbolic and material exclusion from full participation within society (Ferguson, 2001; Johnson-Bailey, 2002; Bell, 2004).

## Conclusion

This racialized exclusions of Black men and boys across important social venues in United States society through negative symbolic framing and enacted practices typically has profoundly negative, harmful implications for their lives and life chances (Shujaa, 1996; Lipman, 1998; Kunjufu, 2001; Sullivan and Cross, 2016). As a consistent consequence of the negative symbolic framing and enacted practices targeting Black men and boys, they regularly face challenges related to lower levels of educational attainment, higher rates of mental illness, higher rates of substance use disorders, higher rates of incarceration, and a host of other social problems (Moody, 2001; Waxman, 2021).

Given the role of symbolic and actual, or physically enacted, negative framing and practices (e.g., Guthrie, 2003) applied to Black men and boys (Haller, 1995; Holtzman, 2000), it is important to challenge these negative images and patterns of acting, interacting, and decision-making which together regularly work to symbolically, and physically, incarcerate them and limit their life chances (Hooks, 2004a; Williams et al., 2022). To create a more just and inclusive society where all people, regardless of race or ethnicity, gender, sexuality and more, experience inclusive, safe, and supportive contexts to foster and further the development of their full potential, it is important and necessary to interrupt the bad faith that connects the freedom-unfreedom paradox they consistently face across institutions (Grier, 2020; Brown, 2021). By exposing the bad faith that connects the symbolic and actual practices which consistently contain Black men and boys (Jackson, 2011), it becomes possible to identify possibilities for positive, transformative strategies and points of intervention required to foster the development of each person’s fully humanity, potential and wellbeing (Hooks, 2000/2018; Matthew, 2015; Wade, 2021).

## Data availability statement

The original contributions presented in the study are included in the article/supplementary material, further inquiries can be directed to the corresponding author.

## Author contributions

All authors listed have made a substantial, direct, and intellectual contribution to the work and approved it for publication.

## References

[ref1] Alexander M. (2010/2020). The New Jim Crow: Mass incarceration in the Age of Colorblindness (10th Edn.). New York: The New Press.

[ref2] Arendt H. (2020). The Freedom to be Free. New York: Penguin Books.

[ref3] Baldwin J. (1962/2021). The Price of the Ticket. New York: Beacon Press.

[ref4] Bell D. (2004). Silent Covenants: Brown v. Board of Education and the Unfulfilled Hopes for Racial Reform. New York: Oxford University Press.

[ref5] Benjamins M. R. De Maio F. G. (eds.). (2021). Unequal Cities: Structural Racism and the Death Gap in America’s Largest Cities. Baltimore: John Hopkins University Press.

[ref6] Bonilla-Silva E. (2003). Racism Without Racists: Color-Blind Racism and the Persistence of Racial Inequality in the United States. New York: Rowman & Littlefield.

[ref7] Bottiani J. H. Bradshaw C. P. Gregory A. (2018). Nudging the gap: introduction to the special issue “closing in discipline disproportionality”. Sch. Psychol. Rev. 47, 109–117. doi: 10.17105/SPR-2018-0023.V47-2

[ref8] Boyd T. M. (2009). Confronting racial disparity: legislative responses to the school-to-prison pipeline. Harvard Civil Rights Civil Libert. Law Rev. 44, 571–580.

[ref9] Bradshaw C. P. Mitchell M. M. O-Brennan L. M. Leaf P. J. (2010). Multi-level exploration of factors contributing to the overrepresentation of black students in office disciplinary referrals. J. Educ. Psychol. 102, 508–520. doi: 10.1037/a0018450

[ref10] Brinton D.G. (1890). Races and People. New York: N.D.C. Hodges.

[ref11] Brook B. (1999). Feminist Perspectives on the Body. London and New York: Longman.

[ref12] Brown Y. (2021). Black Men, Mental Health, and Oppression: What Do We Learn When We Listen to Black Men’s Voices? New York: Coventry University Press.

[ref13] Bureau of Justice Statistics (2014). Recidivism of prisoners released in 30 states in 2005: Patterns from 2005 to 2010. US Department of Justice, Washington DC.

[ref14] Cook C. R. Duong M. T. Pullmann M. McIntosh K. McGinnis J. Fiat A. E.. (2018). Addressing discipline disparities for black male students: linking malleable root causes to feasible and effective practices. Sch. Psychol. Rev. 47, 135–152. doi: 10.17105/SPR-2017-0026.V47-2

[ref15] Cottman M. H. Gaines P. Bunn C. Charles N. Harriston K. (2023). Say Their Names: How Black Lives Came to Matter in America. New York: Grand Central Publishing.

[ref16] Croteau D. Hoynes W. (2014). Media/Society: Industries, Images, and Audience (5th Edn.). New York: Sage Publications.

[ref17] Dean C. (2000). Boys and girls: popular depictions of African American children and childlike adults in the United States 1850-1930. J. Am. Comp. Cult. 23, 17–20.

[ref18] Delpit L. (1995). Other People’s Children: Cultural Conflict in the Classroom. New York: New Press.

[ref19] Dodson N. A. (ed.). (2021). Adolescent Gun Violence Prevention: Clinical and Public Health Solutions. Switzerland: Springer Publishing.

[ref20] DuBois W. E. B. (1899/2023). The W. E. B. DuBois Collection. New York: Grapevine India.

[ref21] Fanon F. (2013). Black Skin, White Masks. New York: Pluto Press.

[ref22] Feagin J.R. (2006). Systemic Racism: A Theory of Oppression. New York: Routledge.

[ref23] Feagin J. R. (2020). The White racial Frame: Centuries of Racial Framing and Counter-Framing (3rd Edn.). New York: Routledge.

[ref24] Feagin J. R. Ducey K. (2018). Racist America: Roots, Current Realities, and Future Reparations (4th Edn.). New York: Routledge.

[ref25] Ferguson A. A. (2001). Bad Boys: Public Schools and the Making of Black American Masculinity. Ann Arbor, MI: The University of Michigan Press.

[ref26] Ferreira da Silva D. (2009). No-Bodies. Griffith Law Rev. 18, 212–236. doi: 10.1080/10383441.2009.10854638

[ref27] Foner E. (1999). The Story of American Freedom. New York: W. W. Norton & Co.

[ref28] Fuller M. L. (1992). Monocultural teachers and multicultural students: a demographic clash. Teach. Educ. 4, 87–93. doi: 10.1080/1047621920040210

[ref29] Glaude E. S. Jr. (2017). Democracy in Black. New York: Crown Publishing.

[ref30] Gordon L. (1995). Bad Faith and Antiblack Racism. New York: Humanity Books.

[ref31] Gordon L. (ed.) (1997). Existence in Black. New York: Routledge.

[ref32] Gossett T. F. (1997). Race: The New History of an Idea in America. New York: Oxford University Press.

[ref33] Grant D. E. (2019). Black Men, Intergenerational Colonialism, and Behavioral Health: A Noose across Nations. Cham, Switzerland: Palgrave Macmillan.

[ref34] Grier N. (2020). Care for the Mental and Spiritual Health of Black Men: Hope to Keep Going. New York: Lexington Books.

[ref35] Guthrie R. V. (2003). Even the Rat Was White: A Historical View of Psychology (2nd Edn.). Boston: Allyn & Bacon.

[ref36] Hale J. E. (2001). Learning While Black: Creating Educational Excellence for African American Children. Baltimore: John Hopkins University Press.

[ref37] Hall S. (ed.). (1997). Representation: Cultural Representations and Signifying Practices. Thousand Oaks, CA: Sage Publications.

[ref38] Haller J.S. (1995). Outcasts From Evolution: Scientific Attitudes of Racial Inferiority, 1859–1900. Carbondale, IL: Southern Illinois University Press.

[ref39] Holtzman L. (2000). Media Messages. Armonk, NY: M.E. Sharpe.

[ref40] Hooks B. (2000/2018) All About Love: New Visions. New York: William Morrow and Company, Inc.

[ref41] Hooks B. (2004a). We Real Cool: Black American Men and Masculinity. New York: Routledge.

[ref42] Hooks B. (2004b). The Will to Change: Men, Masculinity, and Love. New York: Washington Square Press.

[ref44] Irvine J. J. (1990). Black American Students and School Failure: Policies, Practices, and Prescriptions. Westport, CT: Greenwood.

[ref45] Jackman M. (1986). Some of my best friends are black American: interracial friendship and Whites' racial attitudes. Public Opin. Q. 50, 459–486. doi: 10.1086/268998

[ref46] Jackson C. (2011). Violence, Visual Culture, and the Black Male Body. New York: Routledge.

[ref47] Johnson-Bailey J. (2002). Race matters: the unspoken variable in the teaching-learning transaction. New Direct. Adult Contin. Educ. 2002, 39–50. doi: 10.1002/ace.48

[ref48] Jordan W. D. (1974). The White Man’s Burden. New York: Oxford University Press.

[ref49] Kaiser Family Foundation (2006). Race and Ethnicity Health Care Fact Sheet. Washington, DC: The Henry J. Kaiser Family Foundation.

[ref50] Karenga M. (2002). (3rd Edn. ed.). Introduction to Black American Studies. Los Angeles: University of Sankore Press.

[ref51] Kelley R. (1997). Yo Mama’s Disfunktional! Boston: Beacon Press.

[ref52] Kim C. Y. Losen D. J. Hewitt D. T. (2010). The School-to-Prison Pipeline: Structuring Legal Reform. New York, NY: New York University Press.

[ref53] King J. E. (2005). Black American Education: A Transformative Research and Action Agenda for the New Century. Mahwah, NJ: Lawrence Erlbaum Associates.

[ref54] Kunjufu J. (2001). State of Emergency: We Must Save African American Males. Chicago: African American Images.

[ref55] Lipman P. (1998). Race, Class, and Power in the School Restructuring. Albany: State University of New York.

[ref56] Lomotey K. Smith W. A. (eds.) (2023). The Racial Crisis in American Higher Education. (3rd Edn.). Albany: State University of New York Press.

[ref57] Lozenski B. D. (2016). The desirability of discomfort: riding the truth of dissonance in teaching and research. Crit. Questions Educ. 7, 268–286.

[ref58] Matthew D. B. (2015). Just Medicine: A Cure for Racial Inequality in American Healthcare. New York: New York University Press,

[ref60] McIntosh K. Girvan E. J. Fairbanks Falcon S. McDaniel S. C. Smolkowski K. Bastable E.. (2021). Equity-Focused PBIS Approach Reduces Racial Inequities in School Discipline: A Randomized Controlled Trial. Washington, D.C.: American Psychological Association. 10.1037/spq0000466 34766811

[ref61] Mills C. W. (1997). The Racial Contract. Ithaca, NY: Cornell University Press.

[ref62] Montagu A. (1997). Man’s Most Dangerous Myth. Walnut Creek, CA: Alta Mira Press.

[ref63] Moody J. (2001). Race, school integration, and friendship segregation in America. Am. J. Sociol. 107, 679–716. doi: 10.1086/338954

[ref64] Moody-Ramirez M. Tait G. B. Bland D. (2021). An analysis of George Floyd-themed memes. J. Soc. Media Soc. 10, 373–401.

[ref65] Mutua A. D. (ed.) (2006). Progressive Black Masculinities. New York: Routledge.

[ref66] Nicholson C. Birchmeier Z. Valentine D. (2009). Exploring the impact of school discipline on racial disproportion in the juvenile justice system. Soc. Sci. Q. 90, 1003–1018.

[ref67] Noguera P. A. (2003). Schools, prisons, and social implications of punishment: rethinking disciplinary practices. Theory Pract. 42, 341–350. doi: 10.1207/s15430421tip4204_12

[ref68] Patterson O. (2018). Slavery and Social Death: A Comparative Study. Cambridge, MA: Harvard University Press.

[ref69] Pettit B. Gutierrez C. (2018). Mass incarceration and racial inequality. Am. J. Econ. Sociol. 77, 1153–1182. doi: 10.1111/ajes.12241, PMID: 36213171 PMC9540942

[ref70] Pierce L. Heitz K. (2020). Say their names. Am. Q. 72, 961–977. doi: 10.1353/aq.2020.0054

[ref71] Reed E. Lawrence Santana D. A. Welles M. C. Horsburgh S. L. C. Silverman R. C.. (2014). Adolescent experiences of violence and relation to violence perpetration beyond young adulthood among an urban sample of black American and African American males. J. Urban Health 91, 96–106. doi: 10.1007/s11524-013-9805-z, PMID: 23657905 PMC3907629

[ref72] Rodney W. (1972). How Europe Underdeveloped Africa. Washington, D.C.: HowardUniversity Press.

[ref73] Said E. (2003). Orientalism. New York: Penguin.

[ref74] Shujaa M. J. (1996). Beyond Desegregation: The Politics of Quality in African-American Schooling. Thousand Oaks, CA: Corwin Press.

[ref75] Skiba R. J. Michael R. S. Nardo A. C. Peterson R. (2000). The color of discipline: sources of racial and gender disproportionality in school punishment. Indiana education policy center policy research report #SRS1. Bloomington, Indiana: Indiana Education Policy Center.

[ref76] Smedley A. (1993). Races in North America: Origin and Evolution of a Worldview. Boulder: Westview Press.

[ref77] Smith D. T. (2005). These house negroes think we cursed. Cult. Stud. 19, 439–454. doi: 10.1080/09502380500219456

[ref78] Sullivan J. M. Cross W. E. Jr (eds.) (2016). Meaning-Making, Internalized Racism, and African American Identity. Albany: State University of New York Press.

[ref79] Sum A. Khatiwada I. McLaughlin J. Palma S. (2009). The Consequences of Dropping Out of High School: Joblessness and Jailing for High School Dropouts and the High Cost for Taxpayers. Boston, MA: Center for Labor Market Studies.

[ref80] Szetela A. (2020). Black lives matter at five: limits and possibilities. Ethn. Racial Stud. 43, 1358–1383. doi: 10.1080/01419870.2019.1638955

[ref81] Tichavakunda A. A. (2021). Studying and challenging racism in higher education: naming bad faith to understand the “logic” of racism. Educ. Sci. 11, 1–10. doi: 10.3390/educsci11100602

[ref82] Turner E. A. (2019). Mental Health Among African Americans: Innovations in Research and Practice. New York: Lexington Books.

[ref83] Voelkl K. E. Welte J. W. Wieczorek W. F. (1999). Schooling and delinquency among white and African American adolescents. Urban Educ. 34, 69–88. doi: 10.1177/0042085999341005

[ref84] Wacquant L. (2000). Deadly symbiosis: when ghetto and prison meet and mesh. Punishment Soc. 3, 95–133. doi: 10.1177/14624740122228276

[ref85] Wade B. (2021). Grieving While Black: An Anti-racist Take on Oppression and Sorrow. Berkeley, CA: North Atlantic Books.

[ref86] Watkins M. (2006). Stepin Fetchit: The Life and Times of Lincoln Perry. New York: Vintage Books.

[ref87] Waxman S. (2021). Racial awareness and Bias begin early: developmental entry points, challenges, and a call to action, perspectives in psychological. Science 16, 893–902. doi: 10.1177/17456916211026968 34498529

[ref89] Weissinger S. E. Mack D. A. Watson E. (eds.) (2017). Violence Against Black Bodies: An Intersectional Analysis of How Black Lives Continue to Matter. New York: Routledge.

[ref90] Wilderson F. B. (2020). Afropessimism. New York: Liveright Publishing Corporation.

[ref91] Williams M. T. Faber S. C. Duniya C. (2022). Being an anti-racist clinician. Cogn. Behav. Therap. 15, 1–22. doi: 10.1017/S1754470X22000162

[ref92] Yancy G. (ed.) (2022). Black Men from Behind the Veil: Ontological Interrogations. New York: Lexington Books.

[ref93] Yancy G. A. Alcoff L. M. (2016). Black Bodies, White Gazes: The Continuing Significance of Race in America (2nd Edn.). New York: Roman & Littlefield Publishers.

[ref94] Zeng Z. Minton T. D. (2021). Jail inmates in 2019. Bureau of Justice Statistics, Office of Justice Programs, U.S. Department of Justice, Washington, D.C.

[ref95] Zimring F. E. (2017). When Police Kill. Cambridge, MA: Harvard University Press.

